# Embracing the Future of Surgery: Gastric Cancer Resection Within One Month of Left Ventricular Assist Device Implantation

**DOI:** 10.7759/cureus.2868

**Published:** 2018-06-23

**Authors:** Elbrus Zarbaliyev, Mehmet Balkanay, Dauren Sarsenov

**Affiliations:** 1 General Surgery, Istanbul Yeni Yuzyıl University/Gaziosmanpasa Hospital, Istanbul, TUR; 2 Cardiovascular Surgery, Istanbul Yeni Yuzyıl University/Gaziosmanpasa Hospital, Istanbul, TUR; 3 General Surgery, Altunizade Acibadem Hospital, Istanbul, TUR

**Keywords:** gastric cancer, lvad, proximal gastrectomy

## Abstract

Left ventricular assist devices (LVADs) have been implanted recently, with increasing frequency, to treat advanced heart failure with good survival rates. Since heart failure is most prevalent in patients above 70 years of age, LVAD implantations are increasing particularly in this cohort. On the other hand, due to a higher incidence of malignant tumors in the elderly population, there is a significant cohort of patients having concurrent indications for LVAD implantation.

Herein, we report a case of complicated gastric malignancy that was encountered soon after the implantation of an emergent LVAD with ensuing treatment difficulties and ethical considerations.

Keeping in mind the fairly high life expectancy for both groups, there is a predisposition to the notion that simultaneous procedures can and should be applicable to a selected group of patients with end-stage heart failure.

## Introduction

Heart failure prevalence is increasing rapidly due to aging population worldwide, and it is estimated that several millions of people are in the terminal phase of heart failure around the globe [1]. Following the diagnosis of end-stage heart failure, a mortality rate of 20% is expected each year [2]. As results of the landmark Randomized Evaluation of Mechanical Assistance for the Treatment of Congestive Heart Failure (REMATCH) trial became available, demonstrating a superior outcome favouring left ventricular assist devices (LVAD) over best medical therapy in terms of survival, these devices are being recommended for patients diagnosed with end-stage heart failure and awaiting heart transplant [3]. Since then, the indications for LVAD implant have expanded to include three area namely bridge to transplant, bridge to candidacy, and destination therapy [2]. Given these benefits, LVAD implantation rates continue to rise each year, along with an increasing need for non-cardiac surgery (NCS) in these patients [4-6]. Cardiac-related problems occur in about a fourth of gastrointestinal malignancy patients; however, only 4% of patients with a cardiac-related disease are diagnosed with a gastrointestinal malignancy [3]. The following case represents an extreme example of how far this need has expanded over the years with reliable outcomes provided with these devices.

## Case presentation

We report the case of a 64-year-old male whose medical history included hypertension, type II diabetes mellitus, and ischemic dilated cardiomyopathy with a history of heavy smoking (2 package/day for 30 years). The patient had undergone a coronary artery bypass grafting 10 years ago.

**Figure 1:**
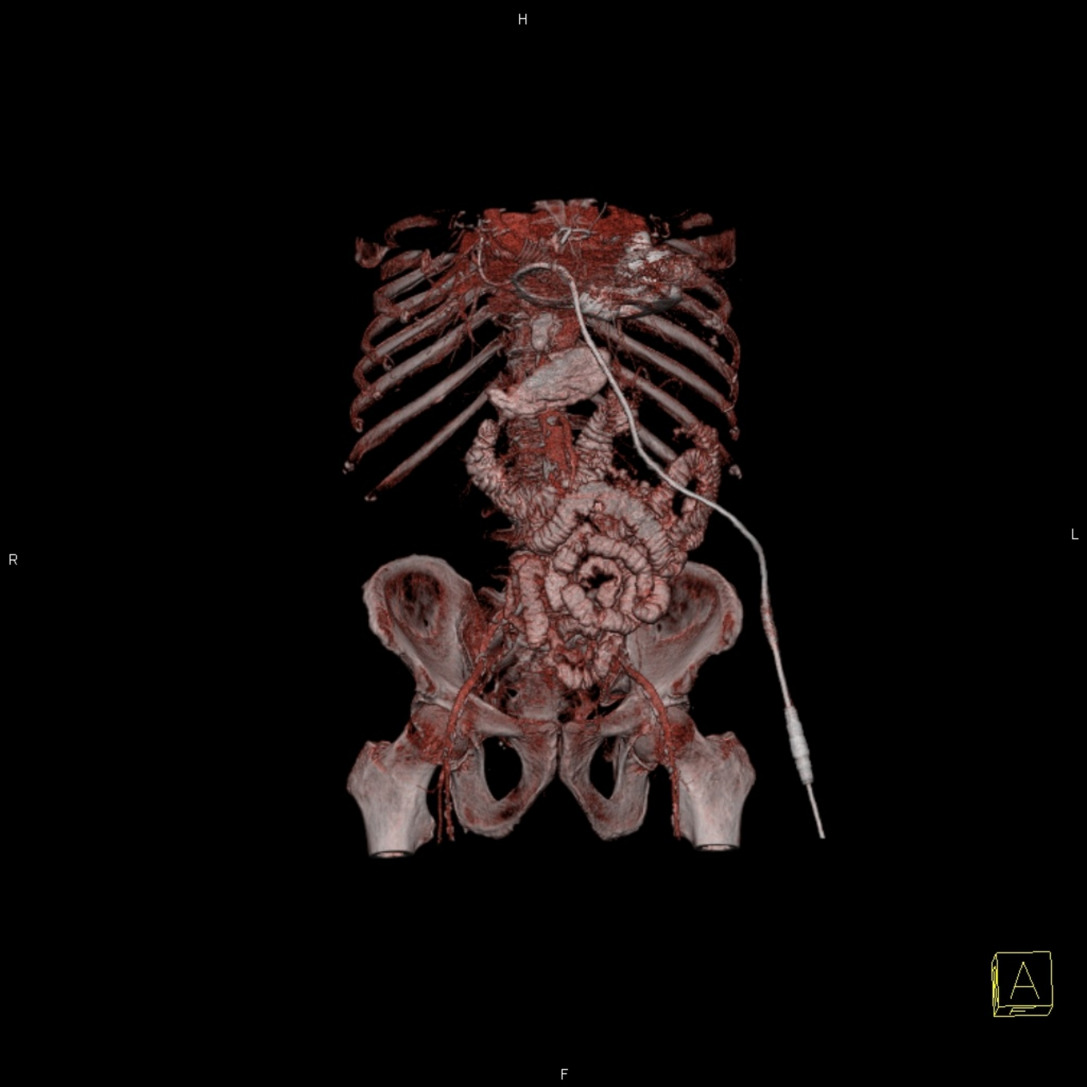
Reconstructed left ventricular assist device (LVAD) image Reconstructed three-dimensional image of the abdominal cavity with a depiction of LVAD surrounding the ventricles.

While being on routine outpatient follow-up due to suddenly worsening heart failure, with signs of systemic edema resulting in dyspnea, extreme fatigue and hypotension, the patient was categorized to have Interagency Registry for Mechanically Assisted Circulatory Support (INTERMACS) II effort capacity. Further need for serious inotropic support placed the patient in the 'bridge to transplant', status Ib category. Consequently, the patient was implanted with a centrifugal type flow LVAD (Heartmate III, St. Jude Medical, Abbott) and was started on anticoagulation therapy with enoxaparin sodium 6000 IU subcutaneously twice daily (Figure 1).

At post-operative day 20, he presented with melena accompanied with haemoglobin (Hb) levels falling from 11 to 6.4 g/dL, which prompted an upper gastrointestinal endoscopy revealing gastric cancer located at the cardia starting 1.5 cm distal to the Z-line, protruding into the gastric lumen at the posterior wall. Due to the patient's high-risk cardiovascular condition, enoxaparin was maintained at the therapeutic level twice daily at 6000 IU.

**Figure 2:**
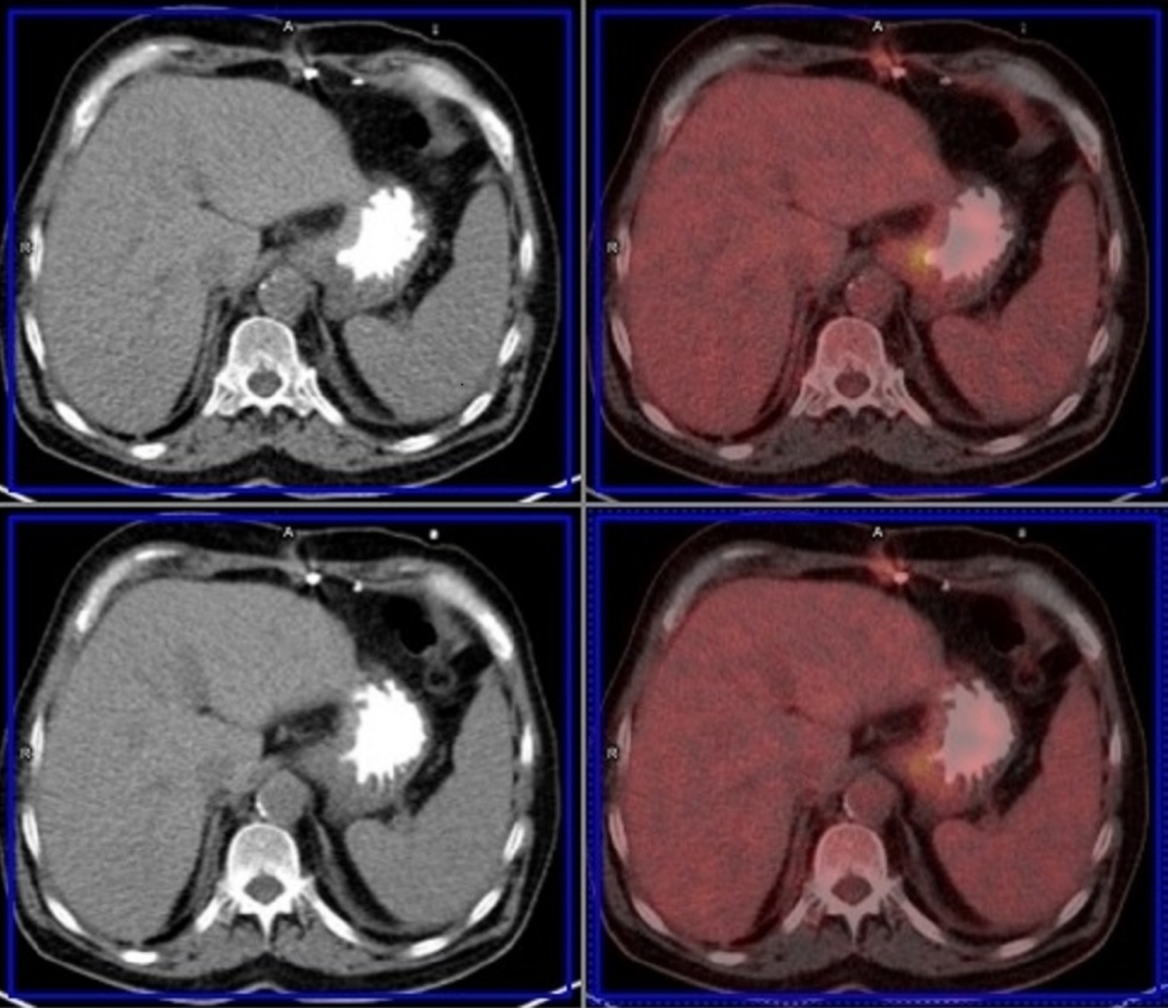
Fusion images of the gastric tumor Gastric tumor involving the posterior wall with elevated ^18^fluorodeoxyglucose metabolism in fusion images on positron emission tomography-computed tomography (PET-CT).

Biopsy indicated a signet-ring cell adenocarcinoma which with subsequent positron emission tomography-computed tomography (PET-CT) demonstrated no distant organ metastasis (Figure 2).

After necessary preoperative assessment by anaesthesiologists and cardiologists, and under full therapeutic anticoagulation protocol (enoxaparin sodium 6000 IU twice daily), it was decided to proceed with a proximal gastric resection due to potentially life-threatening upper gastrointestinal haemorrhage. Since the LVAD was implanted at the left upper quadrant, an upper midline incision was found feasible, and proximal gastrectomy with extended D1 dissection was then performed. We performed resection of the proximal stomach using linear staplers, with a consecutive application of end-to-end circular 28 mm stapler for end-to-side esophagogastrostomy performed on the anterior surface of the stomach. Choice of this particular technique was justified due to the significantly shorter duration (20 minutes for resection-dissection-anastomosis) of the procedure in the light of having a shorter suture row on fundus and one anastomosis to deal with instead of three separate suture rows/anastomoses for Roux-en-Y reconstruction after total gastrectomy.

The pathology report indicated a poorly differentiated pT3N2(10/15)M0 signet-ring cell adenocarcinoma with negative resection margins. Originating from esophagogastric junction tumor was revealed to have perineural and lymphovascular invasion.

The early postoperative period was without major complications. The intravenous fluid administration was restricted in order to limit systemic edema. Since no postoperative bleeding occurred, there was no need to adjust the anticoagulation regimen. The patient experienced minor superficial surgical site infection and was discharged home on postoperative day 11 on a normal diet with necessary adjustments. No major complications were seen. Since then the patient has been followed-up for nine months till date, perfectly tolerating a normal diet.

## Discussion

LVADs are being implanted for advanced heart failure with an increasing frequency, and it is expected that costs will drop accordingly given the advances in technology. The need for NCS following LVAD implants is on the rise given the favourable survival results. Approximately 15%-30% patients require NCS due to various indications, profoundly related to surgical emergencies including appendicitis or cholecystitis [1,3-4,6-7].

The oldest data available in literature encompassed two major operations, one nephrectomy due to renal cell carcinoma and one lower lung lobe resection due to empyema [7]. This was followed by another case series which reported on two lung resections as major operations, of which one was fatal [6]. A recent report demonstrated that NCS did not alter survival in patients implanted with an LVAD, which was reported to be in the range of 56%-64%. There were two colectomy/colostomy procedures performed for diverticulitis, and none of the NCS undertaken was related to malignancy [3].

Stehlik et al. reported a case series including 59 NCS patients; colon resection due to unspecified diagnosis was undertaken in one case where the patient succumbed due to multi-organ failure at postoperative day 21 [4]. Garatti et al. reported 12 NCS cases performed in a cohort of 77 patients receiving LVAD implants, which included one right colectomy performed for adenoma [1]. The only successful case of gastric resection performed for malignancy following LVAD implantation till date was reported by Nakamura in 2017. Total gastrectomy was performed for early gastric cancer involving the anterior wall of the corpus, which was complicated by a duodenal stump blowout [8].

Since the literature is severely limited, a cost-efficiency analysis is not possible owing to the unavailable data. Currently, LVAD implantation in a patient who is newly diagnosed with a malignancy is not recommended [9]. It is recommended to consider patients with a history of malignancy of at least five years negative follow-up in order to indicate a bridge to candidacy for LVAD implantation. Nevertheless, in the present case, LVAD was implanted on an urgent basis and was followed by a gastric malignancy which was diagnosed within 20 days of implantation, and a successful gastric resection was performed. The urgent nature of this case did not leave room for another option other than to operate on the bleeding gastric cancer patient. Should this malignancy be diagnosed and operated before the decision of LVAD implantation, would something differ in terms of our approach to the heart failure? We think that technological progress will, as always, make such considerations obsolete in the near future, thereby driving the medical community to another reconsideration of LVAD implantation indications. On the other hand, operating a patient for a malignant disease even without emergent complications with previously implanted LVAD logically should not be a contraindication as end-stage heart failure is the only absolute contraindication for this as LVAD is supposed to compensate heart failure at least temporarily. In our opinion, cost-effectiveness, cross-survival benefits of both procedures for either malignancy, and end-stage heart failure should be the subject of further research.

One of the main considerations regarding the procedure was the choice of resection type. Proximal gastric resection was preferred in order to shorten procedure duration which was meaningful in the light of therapeutic level anticoagulation. Another consideration was to leave as few suture lines as possible. Since there are no published series on gastric resections performed in end-stage cardiac failure patients, we could not gather information on the comparison between proximal and total gastric resection.

To the best of our knowledge, the present case is the second successful gastric cancer resection in an end-stage heart failure patient and the first case of a patient being discharged without a major complication. The patient is alive after ten months of the operation, is in a good condition and tolerating a normal diet.

The implantation technique poses a true challenge due to the obliteration of both upper quadrants. Since the pocket of the pump chamber in the posterior rectal sheath is best to be preserved in order to avoid the risk of device infection, a median laparotomy was ill-advised [6]. In the present case, the implantation site was limited by the left upper quadrant, which permitted a safe upper midline laparotomy.

Given the increased life-span of patients with LVAD implants, the example of the present case is expected only to recur in the near future. Even in extreme situations such as the present case, it seems possible to resect a gastrointestinal malignancy in the immediate postoperative period of an LVAD implantation, as a first line treatment alternative, when confronted. Employing an implantation technique which bears in mind an allowance of a safe upper midline laparotomy has provided us with an opportunity of close-term interventions for consequently diagnosed two fatal illnesses in a row within 30 days of each other. Further evaluation of the present experience on this subject should guide us to clinical recommendations for this unfavourable combination of a malignancy with an end-stage heart failure.

## Conclusions

Life-threatening complications of malignant conditions can be safely managed in patients after LVAD implantation. Further studies should investigate the possibility of expanding the indications for both the implantation of LVAD in selected cancer patients and the surgical treatment of patients with newly diagnosed malignant disease after LVAD implantation.
